# Effect of Artificial Solar Radiation on the Die-Off of Pathogen Indicator Organisms in Urban Floods

**DOI:** 10.1007/s41742-018-0160-5

**Published:** 2018-11-27

**Authors:** I. M. Scoullos, C. M. Lopez Vazquez, J. van de Vossenberg, M. Hammond, D. Brdjanovic

**Affiliations:** 1 Department of Biotechnology, Delft University of Technology, Van der Maasweg 9, 2629 HZ Delft, The Netherlands; 2 Department of Environmental Engineering and Water Technology, IHE Delft Institute for Water Education, P.O. Box 3015, 2601 DA Delft, The Netherlands; 3 Environment and Natural Resources Global Practice, World Bank Group, Washington, DC USA

**Keywords:** Solar inactivation, Urban drainage, Urban floods, Waterborne diseases

## Abstract

In the last decade, flooding has caused the death of over 60,000 people and affected over 900 million people globally. This is expected to increase as a result of climate change, increased populations and urbanisation. Floods can cause infections due to the release of water-borne pathogenic microorganisms from surcharged combined sewers and other sources of fecal contamination. This research contributes to a better understanding of how the occurrence of water-borne pathogens in contaminated shallow water bodies is affected by different environmental conditions. The inactivation of fecal indicator bacteria *Escherichia coli* was studied in an open stirred reactor, under controlled exposure to simulated sunlight, mimicking the effect of different latitudes and seasons, and different concentrations of total suspended solids (TSS) corresponding to different levels of dilution and runoff. While attachment of bacteria on the solid particles did not take place, the decay rate coefficient, *k* (d^−1^), was found to depend on light intensity, *I* (W m^−2^), and duration of exposure to sunlight, *T* (h d^−1^), in a linear way (*k *= *k*_D_+ 0.03·*I* and *k *= *k*_D_+ 0.65·*T*, respectively) and on the concentration of TSS (mg L^−1^), in an inversely proportional exponential way (*k *= *k*_D_+ 14.57·e^−0.02·*[TSS]*^). The first-order inactivation rate coefficient in dark conditions, *k*_D_= 0.37 d^−1^, represents the effect of stresses other than light. This study suggests that given the sunlight conditions during an urban flood, and the concentration of indicator organisms and TSS, the above equations can give an estimate of the fate of selected pathogens, allowing rapid implementation of appropriate measures to mitigate public health risks.

**Electronic supplementary material:**

The online version of this article (10.1007/s41742-018-0160-5) contains supplementary material, which is available to authorized users.

## Article Highlights

- It was demonstrated under controlled conditions that the inactivation of fecal indicator bacteria ***E. coli*** is higher under higher solar irradiance, longer duration of daylight and low TSS concentrations.
- The results indicate that under high TSS concentrations the bacteria, even if not attached on particles, are protected from photo-inactivation for a period of a few days, as the decay rate decreases exponentially with an increase in TSS concentration.

**Electronic supplementary material:**

The online version of this article (10.1007/s41742-018-0160-5) contains supplementary material, which is available to authorized users.

## Introduction

The spread of pathogenic microorganisms by contaminated flood waters is a serious global problem connected with the spread of diseases. In the last 10 years, global floods have caused the death, by physical injury, drowning or infection, of around 63,000 people, almost 158,000 in the last 20 years and affected over 900 million people (EM-DAT 2014). The initial presence or absence of pathogens in flood waters does not provide a reliable direct indication of the risks to human health. The transport and inactivation of the pathogens appear to play a crucial role in defining public health risks since most of the outbreaks of diseases occur within different time intervals after flooding events (Du et al. 2010; Taylor et al. 2011; Alderman et al. 2012). Several studies have shown that after a flood event, water-borne pathogens from humans and animals are commonly present on the flooded surfaces (ten Veldhuis et al. 2010; Rui et al. 2018). For example, in the Bellamyplein water plaza in Rotterdam, *Campylobacter* was found in high concentrations in the flood water, posing significant health risks (Sales-Ortells and Medema 2015). Despite the obvious importance of the issue, most studies until now were based on case-specific events, making it very difficult to have a broader understanding of the mechanisms of transport and inactivation of pathogens. To obtain a more realistic account of the overall health risks, it is necessary to assess the critical factors influencing the viability of pathogenic microorganisms in the urban environment under different environmental conditions.

Sunlight has been recognised as one of the predominant factors leading to the inactivation of enteric microorganisms in surface waters (Davies-Colley et al. 1999). The sensitivity of microorganisms to electromagnetic radiation depends on the wavelength and on the species of organisms and is described by biological weighting functions (Silverman and Nelson 2016). Although bacteria have greatest sensitivity to short wavelengths of light (Reed 2004), the short wavelength light is faster attenuated in the water column having a smaller fraction of the incident radiation (Silverman and Nelson 2016). Therefore, the lethal effect of sunlight on bacteria is more important in wavelengths higher than 320 nm (Oppezzo 2012). These wavelengths can cause indirect (photo-chemical, particularly photo-oxidative) damage, when absorbed by photosensitiser macromolecules within microbial cells (endogenous, such as porphyrins, bilirubin or chlorophyll) or in the surrounding water (exogenous, such as dissolved organic matter or humic acids) (Maraccini et al. 2016).

Suspended matter in water allows the encasement (adsorption/absorption) of microorganisms, offering protection from environmental stressors, such as grazing, adverse hydrodynamic conditions and irradiance, by offering shading (refraction, reflection and/or scattering). It can also offer increased access to nutrients of the particles or enhance settling. The degree to which particle association can impact light inactivation of enteric microorganisms in surface waters apparently is not yet clear (Walters et al. 2014).

In this study, the effects on the die-off of pathogens under various light conditions and in the presence of different concentrations of suspended solids were examined in an open reactor using a common indicator organism and an artificial light source with a spectrum similar to solar light. The experimental setting allowed to simulate different solar light intensities and durations that can occur at different times of the year and at different places around the globe. These conditions can be applied to all kinds of shallow water bodies, including multifunctional urban flood retention and detention basins often used as sport facilities or playgrounds during dry weather. Contamination could originate from wastewater from surcharged combined sewers, pit latrines, cross connections, open defecation, animal litter, or municipal and livestock operations. The selection of the light intensity and duration parameters was based on the conditions at two different latitudes: the equator and 60°. The equator zone was selected because it gets the maximum light intensity at a stable daytime length of 12 h throughout the year. 60°N and S were selected because of the daylight duration of 6 and 18 h, respectively, in winter and summer solstice in the North and the opposite in the South.

This study provides a simple mathematical description of how bacterial inactivation is affected by the light conditions and the suspended solid concentrations, which can be used to create and/or upgrade models proposed for describing and predicting the fate and dispersion of pathogens leading to public health risks (Yakirevich et al. 2013; Jonsson and Agerberg 2014). Such models can be used as a tool for adaptation to climate change policies and strategies related to forecasting and properly informing key stakeholders and the public on health risks and appropriate measures associated with flooding events.

## Materials and Methods

### Experimental Reactor

Batch experiments took place in an open cylindrical stirred reactor of 40 cm internal diameter and 80 cm height. For each batch, the reactor was filled with 75 L of demineralised water (up to around 60 cm); suspended solids and indicator organisms were added, according to each experimental phase. Continuous stirring at 95 rpm (IKA^®^ dual-speed overhead stirrer, Staufen, Germany) was used to avoid settling. For maintaining the temperature of the reactor adequately constant at around 25 °C (fluctuating between 20 and 28 °C) and to neutralise the heating effect of the lamp, the reactor was placed in a larger tank containing water as a buffer while a ventilator was also employed. Each batch experiment lasted around 1 week, depending on the die-off rate and until the microorganisms were not fully detected.

### Indicator Organisms

*Escherichia coli* ATCC 25922 was chosen as the faecal indicator bacterium of study, because it has been studied thoroughly and can be grown easily under laboratory conditions. Before each batch experiment, *E. coli* was incubated in 1.3% w/v Oxoid CM0001 Nutrient Broth (Oxoid Ltd., Basingstoke, United Kingdom) solution in Erlenmeyer flasks for 24 h at 37 °C. After incubation, the concentration of the inoculum was around 3·10^9^ CFU mL^−1^. The inoculation took place in the reactor by adding 280 mL of inoculum in demineralised water to achieve an initial concentration of *E. coli* of around 10^7^ CFU mL^−1^. The initial concentration of each batch was measured by sampling 15 min after the inoculation to allow full mixing. The selection of the initial concentration of 10^7^ CFU mL^−1^ was based on the typical high concentrations of *E. coli* in raw municipal wastewater of around 10^6^ CFU mL^−1^ according to Mark et al. (2015) or 5·10^6^ CFU mL^−1^ when taking into account minor contributions of industrial wastewater (Henze and Comeau 2008).

The enumeration of *E. coli* in the samples was performed by counting the number of colony forming units (CFU) on Chromocult^®^ Coliform Agar (CCA) (Merck KGaA, Darmstadt, Germany) plates after 24 h of incubation at 37 °C. Appropriate tenfold dilution steps in 0.1% peptone physiological salt solution were used. The plates were spread in triplicate.

### Suspended Solids

To study the effects of different total suspended solids (TSS) concentrations, soil was used obtained from a previously flooded excavation site in Delft, The Netherlands. The soil was dried, ground and sieved. The particles selected for the experiment after sieving varied between sizes of 38 and 106 μm (silt and fine sand). This fraction corresponds to frequently found particle sizes in urban environments, for example around 28% of the particle size distribution that was measured in a parking lot and at a residential basin (Selbig et al. 2016) and to around 50% of particles measured in asphalt road runoff (Charters et al. 2015). Subsequently, the solids were heated in a muffle furnace at 520 °C for 6 h to remove all organic compounds. The solids were added in the reactor in each batch experiment prior to the inoculation. The studied TSS concentrations varied from 0 to 200 mg L^−1^, because 200 mg L^−1^ is the reference value of US EPA for event mean concentration of TSS for urban runoff, while the similar ones according to NURP and to USGS and NPDES are lower, at 174 and 78.4 mg L^−1^, respectively (Acharya et al. 2010). The measurement of TSS was performed in triplicate with vacuum filtration using Whatman^®^ Grade 1 (GE Healthcare, Little Chalfont, UK) filter papers (11 μm pore size), so that solid particles with attached bacterial cells are retained while non-attached bacteria can pass through. Prior and after the filtration, the filter papers were dried at 105 ^°^C for 2 h and the weight was determined.

### Light Source and Parameters

Simulated sunlight was produced using an OSRAM HQI-BT 400 W/D PRO metal halide lamp with built-in UV filter at around 320 nm. Apart from a few peaks in the area of visible light (with the highest one at 540 nm), the spectrum of these metal halide lamps is continuous (Calin and Parasca 2008). The light intensity was regulated by changing the distance of the light source from the water surface. The duration of light/darkness hours was set with a timer. The photon flux, in μmol m^−2^ s^−1^, was measured with a LI-250A Light Meter equipped with an underwater quantum sensor (LI-COR^®^ Biosciences, Inc., Lincoln, Nebraska, USA). This sensor has a uniform sensitivity in the range of wavelengths between 400 and 700 nm; this was taken into account when calculating the lamp irradiance spectrum. The measurements were performed in duplicate, in different sides of the reactor. The light intensity, in W m^−2^, was calculated with the Planck relation, taking into account the spectral power distribution of the lamp. More details on this are provided in the SI.

### Experimental Design

#### Assessment of Light Attenuation

To address the phenomenon of light attenuation and calculate the light attenuation coefficient in the different conditions of the experiments, the photon flux was measured at different depths, with increments of 5 cm, in the reactor filled with demineralised water inoculated with *E. coli* and with the addition of soil particles of different concentrations (0, 25, 50, 100, 150 and 200 mg TSS L^−1^).

#### Effect of light intensity, duration of exposure to light and different concentrations of TSS on the viability of indicator organisms

The effects of different light intensities (190 and 320 W m^−2^, for 12 h day^−1^, without any solids added), periods of exposure to light (0, 6, 12, 18 and 24 h day^−1^, under 320 W m^−2^, without addition of any solids) and different concentrations of TSS (0, 25, 50, 100, 150 and 200 mg L^−1^, for continuous exposure to light of 320 W m^−2^ for 24 h day^−1^) on the die-off of the indicator organisms in demineralised water were studied. The viability of the organisms was also studied in dark conditions as a control.

#### Comparison of results by testing the inactivation of indicator organisms in artificial floodwater

The inactivation of *E. coli* was studied in artificial floodwater. Domestic wastewater was used to mimic flood water. It was diluted ten times with demineralised water to simulate dilution by storm water runoff. The wastewater was collected from the influent stream of Harnaschpolder wastewater treatment plant, Den Hoorn, The Netherlands, after the first screening filters. Before dilution, the wastewater had a concentration of 29.6 mg TSS L^−1^ and 8.5·10^4^*E. coli* CFU mL^−1^. To study separately the effect of the wastewater’s suspended solids, the experiment was performed by removing the wastewater’s TSS with filtration using Whatman^®^ GF/C (GE Healthcare, Little Chalfont, UK) filters (1.2 μm pore size) and replacing the TSS with 150 mg L^−1^ of the treated soil particles, as described before. *E. coli* was added in the reactor prior to the experiment to achieve an initial concentration of 10^7^ CFU mL^−1^. Wastewater quality has a high variability and the dilution factor from storm water increases the uncertainty even more. Therefore, the scope of this paper is limited in comparing demineralised water with diluted wastewater, both with the addition of soil particles. A comparison is made between the reported characteristics of flooding water and the synthetic flood water used in this research (see Table S1).

### Sampling and physicochemical parameters of study

Samples of 20 mL were taken from the reactor at periodic intervals: (1) approximately, every 12 h when the light was on (2) at the beginning and end of each dark and light phase during the experiment of light duration, and (3) every 24 h when the decay was slow. The samples were taken from the top of the reactor with a 30 mL syringe. For the experiments involving suspended solids where it was important to assess stirring efficiency and sedimentation, samples were also taken from the middle and close to the bottom of the reactor by connecting a metallic tube to the syringe. The measurement of pH took place at the same intervals, with a handheld pH meter (WTW pH 323, WTW GmbH, Weilheim, Germany). Temperature and dissolved oxygen were measured every hour (fluctuating between 20 and 28 °C and between 6.5 and 8.5 mg O_2_ L^−1^, respectively, data not shown) with a handheld DO-meter (WTW Oxi 3310, WTW GmbH, Weilheim, Germany).

### Data Analysis

Light attenuation due to different concentrations of suspended solids was addressed by measuring the vertical distribution of light in air and water with different concentrations of solids. This was described using an exponential function equivalent to Beer’s law (Eq. 1), which states that the light intensity decreases exponentially as a function of depth:

1 $$I_{ (z )} = I_{{ ( {\text{surface)}}}}\cdot e^{ - \mu z}$$

where *z* is the depth (m), *μ* is the light attenuation coefficient (m^−1^), *I*_(*z*)_ is the light intensity (W m^−2^) at depth *z* and *I*_(surface)_ is the light intensity on the surface (*z *= 0 m) (Kirk 1994; Morris et al. 1995).

In all the experiments where the inactivation of *E. coli* was addressed, the decay rates were calculated using the first-order exponential decay “Chick–Watson” model (Eq. 2):

2 $$C\left( t \right) = C_{0}\cdot e^{ - kt}$$

where *t* is the time (d), *C(t)* is the measured concentration of *E. coli* (CFU mL^−1^) at time *t*, *C*_*0*_ is the initial concentration of *E. coli* measured in the reactor at time 0 and *k* (d^−1^) is the first-order decay rate constant. In all experiments, the inactivation was considered to start at time 0 with no lag period. Time 0 was considered as 15 min after inoculation to allow full mixing in the reactor before measuring *C*_*0*_. For each batch experiment, the first-order decay rate was assumed to be constant.

Maraccini et al. (2016) and Davies-Colley et al. (2000) used the following linear model (Eq. 3) to express the total inactivation under light conditions as the sum of the photo-inactivation plus the “dark” inactivation, due to stresses other than light, such as osmotic stress, predation and starvation:

3 $$k = k_{\text{D}} + k_{\text{L}} \cdot I$$

where *k*_D_ is the first-order dark inactivation rate constant (d^−1^), *k*_L_ is a pseudo-second-order photo-inactivation rate constant (m^2^ W^−1^ d^−1^) and *I* is the light intensity (W m^−2^). In the present study, a similar expression was also presented for the relation of *k* to the duration of exposure to light (*T*). Subsequently, *k*_D_ was calculated experimentally as the mean of the values corresponding to the intercept of linear regressions for zero intensity and exposure to light.

## Results and Discussion

### Light Attenuation

The vertical distribution of light intensity followed an exponential pattern, which corresponds to Beer’s law, with values of *R*^2^ > 0.98 for all the different TSS concentrations studied in the reactor (data is presented in Fig. S2). The light attenuation coefficient was proportional to the concentration of suspended solids and Eq. 4 was determined from a linear regression with *R*^2^ = 0.94:

4 $$\mu = 2.59 + 0.02\cdot{\textit{[TSS]}}$$

where *μ* is the light attenuation coefficient (m^−1^) and *[TSS]* is the concentration of total suspended solids (mg L^−1^). The minimum theoretical value of *μ* corresponding to pure water is 2.59 m^−1^.

### Die-Off of *E. coli* Under Different Light Intensities

The die-off of *E. coli* was studied in the reactor under exposure to light for 12 h day^−1^ followed by 12 h in dark, without any solids added. The light intensities studied were 190 and 320 W m^−2^, with a control of 24 h in dark. The inactivation was described with exponential decay kinetics and the resulting decay rates are presented in Table 1. The decay rate increases with an increase in light intensity and the data fit well (*R*^2^ = 0.99) to the linear Eq. 3 with *k*_D_= 0.19 d^−1^ and *k*_L_= 0.03 m^2^ W^−1^ d^−1^.

**Table 1:** Decay rates of *E. coli* in the reactor without addition of any solids for different light intensities (with *T *= 12 h of light per day) and for different periods of exposure to light (with *I *= 320 W m^−2^)

Different light intensities (*I*)		Different periods of exposure to light (*T*)	
*I* (W m^−2^)	*k* (d^−1^) (*R*^2^)	*T* (h)	*k* (d^−1^) (*R*^2^)
0 (dark control)	0.08 (0.76)	0 (dark control)	0.04 (0.47)
190	5.66 (0.86)	6	5.07 (0.92)
320	8.91 (0.92)	12	8.91 (0.92)
		18	10.96 (0.95)
		24 (light control)	16.59 (0.99)

### Die-Off of *E. coli* Under Different Periods of Duration of Exposure to Light

The die-off of *E. coli* was studied in the reactor, without any solids added, under exposure to light of 320 W m^−2^ for 0, 6, 12, 18 and 24 h day^−1^ followed by 24, 18, 12, 6 and 0 h in dark, respectively, per day. The inactivation was described with exponential decay kinetics for the overall curves and the resulting decay rates are presented in Table 1. The decay rate increases with an increase in exposure time and the data fit well (*R*^2^ = 0.98) to the linear Eq. 5:

5 $$k = k_{\text{D}} + k_{\text{L}}^{{\prime }} \cdot T$$

where *k*_D_= 0.54 d^−1^ is the first-order dark inactivation rate constant corresponding to 0 h of exposure to light, *k*_L_^′^ =  0.65 h^−1^ is a pseudo-second-order photo-inactivation rate constant, and *T* is the duration of exposure to light per day (h d^−1^).

### Determination of *k*_D_

The inactivation rate coefficient in dark conditions, *k*_D_, represents the effect of inactivation due to stresses other than light, such as osmotic stress, predation and starvation. Its theoretical value was calculated as the average of the intercepts resulting from the previous two linear regressions, *k*_D_= (0.19 + 0.54)/2 = 0.37 d^−1^. Although experimentally it was measured at a lower value in the dark control experiments conducted in this research (0.08 d^−1^ and 0.04 d^−1^, respectively), the calculated value is broadly consistent with the average values of dark inactivation rates for *E. coli*, of 0.55 d^−1^ (ranging from 0.34 to 0.79 d^−1^) in cold season and 0.79 d^−1^ (from 0.60 to 1.10 d^−1^) in warm season reported by Maïga et al. (2009), with those of 0.48 and 0.55 d^−1^ at average temperature of 20 °C reported by Craggs et al. (2004), 0.41 and 0.55 d^−1^ calculated for waste stabilisation pond and raw sewage in fresh river water, respectively, by Sinton et al. (2002) and with 0.50 d^−1^ at around 24 °C, pH 8.0 and 7.5 mg L^−1^ dissolved oxygen in environmental water reported by Gutiérrez-Cacciabue et al. (2016).

### Die-Off of *E. coli* Under Different Concentrations of TSS

**Fig. 1:**
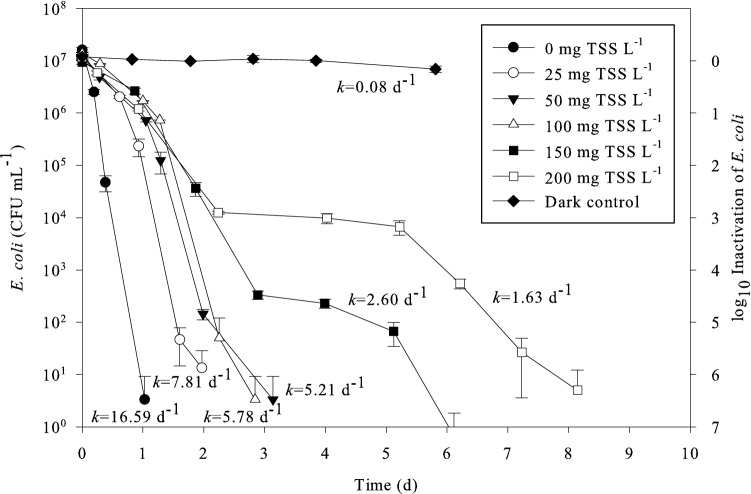
Concentration, log_10_ inactivation and decay rate (*k*) of *E. coli* under exposure to artificial sunlight (320 W m^−2^, 24 h day^−1^) with TSS 0, 25, 50, 100, 150 and 200 mg L^−1^, compared to dark control (no light and no solids). These curves correspond to the samples taken at the top of the reactor prior to filtration

To assess the role of suspended solids in the inactivation of indicator organisms, a series of batch experiments took place using different concentrations of TSS. The results of this experimental phase can be seen in Fig. 1.

Compared to the experiment in absence of suspended solids (*k *= 16.59 d^−1^) it is clear from Fig. 1 that the decay rate decreased with increasing TSS concentration. With absence of suspended solids the decay curve was log_10_-linear, which was not the case in the presence of particles. In fact, in presence of solids, especially over 25 mg L^−1^, there was a one-day slow phase, followed by more rapid inactivation in the next couple of days. For concentrations of 50 and 100 mg TSS L^−1^ the decay curves were almost identical, with decay rates of 5.21 and 5.78 d^−1^, respectively, and with the levels of *E. coli* falling under 10^1^ CFU mL^−1^ before the 4th day of the experiment. The steepest parts of these curves had decay rates of 9.24 d^−1^ for 50 mg TSS L^−1^ and 8.10 d^−1^ for 100 mg TSS L^−1^, which were comparable to the one without solids (16.50 d^−1^) and to that of 25 mg TSS L^−1^ (11.03 d^−1^). For solids’ concentrations of 150 and 200 mg L^−1^ the rapid inactivation decay rates were lower (4.46 and 3.05 d^−1^, respectively), followed by a plateau between day 3 and day 5, before the concentration of indicator organisms started falling more rapidly again, reaching levels below 10^1^ CFU mL^−1^. The *E. coli* bacteria in the experiment came from a pure culture and the experiment was performed in separate batches for 150 and 200 mg TSS L^−1^, with sampling in triplicate; therefore, the observed plateau was obtained in a reproducible manner. Brouwer et al. (2017) also observed such a deviation from the simple exponential decay and modelled that with biphasic decay dynamics (fast decay followed by a phase of slow decay) attributed to population heterogeneity, hardening off, viable-but-not-culturable states and/or density effects. This raised the question of whether the bacteria were eventually attached to the TSS particles. This was examined in the experiments that follow.

**Fig. 2:**
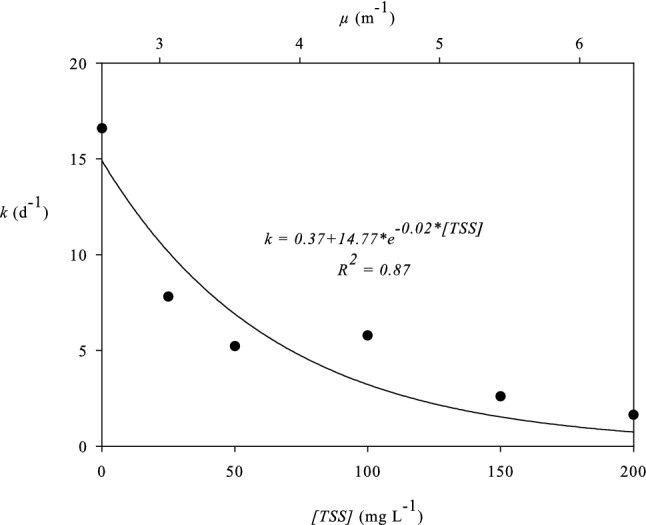
The effect of TSS concentration (mg L^−1^, lower horizontal axis) and of light attenuation coefficient *μ* (m^−1^, upper horizontal axis) on decay rate *k* (d^−1^) of *E. coli* in the reactor under continuous exposure to artificial sunlight (320 W m^−2^). The coefficient *μ* was calculated based on Eq. 4

The decay rates were calculated assuming the traditional model of monophasic decay for the overall period of the experiment, including the lag phase and the plateau, where it exists. The correlation between TSS concentration and overall decay rate (*k*) can be seen in Fig. 2.

The decay rate *k* (d^−1^) decreases with an increase in the concentration of TSS (mg L^−1^) in an exponential way (R^2^ = 0.87) as described by the Eq. 6:

6 $$k = k_{\text{D}} + 14.77\cdot{\text{e}}^{{ - 0.02 \cdot\textit{[TSS]}}}$$

It can be assumed that for very high TSS concentrations light cannot penetrate in the water, so decay rate is similar to that of dark conditions; therefore, the *k*_D_= 0.37 d^−1^ parameter, as calculated previously, has been added to the exponential decay model.

The combination of this with the results of the first experiment leads to the formulation of an exponential relation (Eq. 7) between the decay rate *k* (d^−1^) and the light attenuation coefficient *μ* (m^−1^):

7 $$k = k_{\text{D}} + 65.48\cdot{\text{e}}^{ - 0.65 \cdot \mu }$$

for *μ* ≥ 2.59 m^−1^, which is the minimum theoretical value corresponding to the light attenuation coefficient of water without any solids, as calculated previously. Again, the *k*_D_ parameter has been added because it is assumed that for very high values of *μ* the decay rate is similar to that of dark conditions. This relation can be very useful as an indirect method for predicting the decay rate of *E. coli*-like pathogens in waters by measuring the vertical distribution of light, without the need of measuring the concentration of suspended solids or turbidity. More research on relevant methodologies has been conducted by Nguyen et al. (2015), Silverman and Nelson (2016) and presented in a critical review paper by Nelson et al. (2018).

At this point, it is interesting to note that although the irradiance of the UVA band of wavelengths of this lamp accounts for 5.1% of the total irradiance, as measured from the relative spectral power distribution, biological weighting functions show that the inactivation effect of wavelengths higher than 400 nm on *E. coli* is negligible (Nelson et al. 2018). Therefore, as the lamp has a UV filter at 320 nm, we can assume that the total inactivation caused by this lamp corresponds to the UVA part of the spectrum. The values of the average UVA irradiance transmitted through the water column for the different TSS concentrations studied are presented in Table 2.

**Table 2:** Decay rates of *E. coli* in the reactor with demineralised water (DW) and artificial flood water (FW), at different concentrations of TSS

*[TSS]* (mg L^−1^)	*I* (W m^−2^)	*I*_UVA_ (W m^−2^)	*μ* (m^−1^)	*k* (d^−1^)	*k* (h^−1^)
0 (DW)	156.8	8.0	2.2	16.59	0.69
25 (DW)	125.3	6.4	3.3	7.81	0.33
50 (DW)	85.4	4.4	3.6	5.21	0.22
100 (DW)	65.7	3.4	4.9	5.78	0.24
150 (DW)	57.4	2.9	4.9	2.60	0.11
200 (DW)	37.3	1.9	6.4	1.63	0.07
150 (WW)	53.0	2.7	6.5	4.40	0.18

The average total irradiance, *I*, transmitted through the water column (calculated from Eq. S2), as well as the average UVA irradiance (320–400 nm) transmitted through the water column, *I*_UVA_ = 5.1%*·I*, and the light attenuation coefficient, *μ* (measured values), are also presented

### Attachment and Particle-Related Shielding

The attachment of *E. coli* on the suspended particles and particle-related shielding was tested in a batch experiment in Erlenmeyer flasks in an orbital shaker (150 rpm) with 200 mg TSS L^−1^ of solids in 250 mL of demineralised water by filtering samples of 25 mL and enumerating the bacteria in the filtered and unfiltered samples, in triplicate, at time zero and throughout the duration of the experiment. The soil particles used in the experiment were all selected, using sieving, to be larger than 38 μm and smaller than 106 µm. The bacteria that could have been adhered or attached to particles were retained together with the particles on the filter paper of 11 μm. A t test for paired samples was performed to determine whether the concentrations of *E. coli* before and after filtration are likely to have come from distributions with equal population means. The value of t-statistic (0.93) was lower than the critical value (2.45), so the null hypothesis (that the mean difference in concentration between filtered and unfiltered samples was zero) was accepted. Therefore, it is concluded with 95% confidence that the amount of indicator organisms associated with the suspended solids was not significant.

In addition to that, samples were taken from three different depths in the reactor: close to the surface (2 cm), middle (30 cm) and close to the bottom (58 cm). The concentration of suspended solids was found to be higher near the bottom of the reactor than at the middle and at the top of it because of sedimentation, with some fluctuations over time. The stirring speed was not increased, to avoid the creation of a vortex that would affect the water surface and the uniform distribution of light in the reactor. This inevitably caused the formation of a vertical TSS concentration gradient. Even so, *E. coli* was found to be uniformly distributed at different depths in the reactor (data not shown). This was observed both before and after filtration of the samples, supporting the finding that significant attachment did not occur for any of the TSS concentrations studied.

The absence of irreversible adsorption may be attributed to short incubation time with clean particles, stirring velocity, grain characteristics (size, size uniformity and surface roughness), solution chemistry (zeta potential, ionic strength), geochemical heterogeneity and lipopolysaccharide composition in the bacterial outer membrane (Foppen and Schijven 2006). According to Cantwell and Hofmann (2008), particle association affects the kinetics of inactivation by shielding the bacteria from exposure to light only when the number of particle-associated bacteria is significantly higher than the number of free-floating bacteria. The results indicate that also free-floating bacteria could be protected for some time due to shading under high concentrations of TSS and this is a conclusion of practical importance when strategies are conducted and measures are taken for appropriate protection of population in cases of floods.

### Comparison of Results by Testing the Inactivation of Indicator Organisms in Simulated Floodwater

**Fig. 3:**
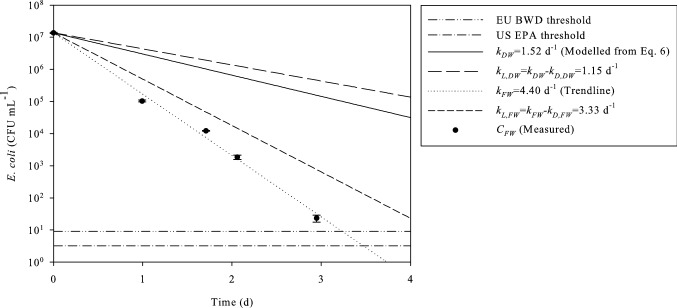
The concentration of *E. coli* in artificial floodwater (*C*_FW_, with decay rate *k*_FW_) measured in the reactor, compared to the theoretical curve for demineralised water with *k*_DW_= 1.52 d^−1^ calculated from Eq. 6 for 150 mg TSS L^−1^. The decay rates without the effect of dark inactivation for the two water qualities (*k*_L,FW_ and *k*_L,DW_, respectively) were calculated by subtracting the dark inactivation rate from the overall decay rate. The curves were plotted using Eq. 2. The threshold concentrations of *E. coli* according to US EPA Recreational Water Quality Criteria and EU Bathing Water Directive are also presented

The decay rate of *E. coli* bacteria in the reactor filled with artificial floodwater containing 150 mg TSS L^−1^ of treated solids and an initial *E. coli* concentration of 1.4·10^7^ CFU mL^−1^, under continuous exposure to light of 320 W m^−2^, was *k*_FW_= 4.40 d^−1^. The predicted value of the decay rate at 150 mg TSS L^−1^ for the same light conditions, according to Eq. 6, would have been *k*_DW_= 1.52 d^−1^. Part of this difference between the predicted and the measured value may be explained by the effect of factors other than light, related to the components of wastewater. Therefore, the experiment was repeated in dark conditions to identify the effect of dark inactivation. In dark conditions, the decay rate in artificial floodwater was *k*_D,FW_= 1.07 d^−1^, which was higher than the one calculated in demineralised water (*k*_D,DW_= 0.37 d^−1^). This confirms that the wastewater, although diluted, had a negative effect on the survival of *E. coli* in dark conditions. The decay rate of inactivation solely due to light, by subtracting the effect of dark inactivation from the overall decay rate, was equal to *k*_L,DW_= *k*_DW _− *k*_D,DW_= 1.15 d^−1^ for demineralised water and *k*_L,FW_= *k*_FW _− *k*_D,FW_= 3.33 d^−1^ for floodwater. The results can be seen in Fig. 3. This means that the negative effect of light was stronger in the floodwater than in demineralised water.

The threshold concentration of *E. coli* according to the EU Bathing Water Directive for sufficient water quality of inland waters is 9 CFU mL^−1^, based on a 90‐percentile evaluation of the log_10_ normal probability density function of microbiological data acquired from the particular bathing water (Directive 2006/7/EC). The 2012 Recreational Water Quality Criteria by US EPA recommend a 90-percentile statistical threshold value of 3.2 CFU mL^−1^ for *E. coli* in fresh water for primary contact recreation, including swimming, bathing, water play by children and similar water activities where a high degree of bodily contact with the water, immersion and ingestion are likely (US EPA 2012). According to the previous results, for an initial concentration of 10^7^ CFU *E. coli* mL^−1^, in floodwater the EU threshold would be met in 4.2 d and the US threshold would be met in 4.5 d if dark inactivation is not taken into account. In demineralised water, the two thresholds will be met only after 12.1 and 13.0 d, respectively.

Although this work provides a better understanding of the die-off of indicator organisms in shallow water bodies, like urban retention and detention basins, further research has to be undertaken employing a wider range of water-borne pathogens and environmental conditions to be able to predict inactivation of pathogens under natural conditions.

## Conclusions

- The light attenuation coefficient obtained in this study, which is proportional to the concentration of suspended solids, is: *μ* = 2.59 + 0.02·*[TSS]*, where *μ* is the light attenuation coefficient (m^−1^) and *[TSS]* is the concentration of total suspended solids (mg L^−1^).
- The decay rate decreases exponentially with an increase in TSS concentration, which can be described in this case as *k *= *k*_D_ + 14.77·e^−0.02·*[TSS]*^, where *k* is the decay rate in d^−1^ and *[TSS]* is the concentration of total suspended solids in mg L^−1^.
- In general, it was demonstrated under laboratory and controlled conditions that inactivation of pathogen indicators *E. coli* is higher under higher solar irradiance, longer duration of daylight and low TSS concentrations.
- The results indicate that under high TSS concentrations the bacteria, even if not attached on particles, are protected from photo-inactivation for a period of a few days.
- It is also noteworthy that the negative effect of light was stronger in the flood water experiments than in those with demineralised water.
- The results can be useful for numerical flood modelling and quantitative microbial risk assessment. Further research is needed, combining real environmental and operating conditions, like temperature and various water quality parameters, as well as with stronger pathogen indicators such as *Cryptosporidium*.

## Electronic supplementary material

Below is the link to the electronic supplementary material.

Supplementary material 1 (DOCX 16 kb)

Supplementary material 2 (EPS 662 kb)

Supplementary material 3 (EPS 1010 kb)

Supplementary material 4 (EPS 955 kb)

Supplementary material 5 (EPS 1171 kb)

Supplementary material 6 (EPS 770 kb)
