# Supplementary material for: Effect of Artificial Solar Radiation on the Die-Off of Pathogen Indicator Organisms in Urban Floods
Source: Int J Environ Res. 2018 Nov 27;13(1):107–16. doi: 10.1007/s41742-018-0160-5 (PMC6383957; doi:10.1007/s41742-018-0160-5)
Supplement: Supplementary file 1 — Supplementary material 1 (DOCX 16 kb) [file 41742_2018_160_MOESM1_ESM.docx]

**Supporting Information**

Effect of artificial solar radiation on the die-off of pathogen indicator organisms in urban floods

**I.M. Scoullos^1*,2^, C.M. Lopez Vazquez^2^, J. van de Vossenberg^2^, M. Hammond^3^, D. Brdjanovic^1,2^**

^1^Department of Biotechnology, Delft University of Technology, Van der Maasweg 9, 2629 HZ Delft, The Netherlands

^2^Department of Environmental Engineering & Water Technology, IHE Delft Institute for Water Education, P.O. Box 3015, 2601 DA , Delft, The Netherlands

^3^Independent Consultant, Crans-près-Céligny, Switzerland

*Corresponding author. Tel.: +31 15 215 1715. E-mail address: i.scoullos@un-ihe.org

**Calculation of light intensity based on photon flux and spectral power distribution**

The total light intensity, *I* (W m^-2^), in the area of wavelengths for which the spectral power distribution of the lamp is provided (320-805 nm), at any location where the sensor is placed, was calculated with the Planck relation, taking into account the spectral power distribution of the lamp, using Equation S1, adapted from Silverman and Nelson (2016):

$$I=\sum_{\lambda=320}^{805} I\left( \lambda\right)=\varphi\cdot h\cdot c\cdot N_{A}\cdot{10}^{3}\cdot\frac{\sum_{\lambda=320}^{805} M\left( \lambda\right)}{\sum_{\lambda=400}^{700} \left[ M\left( \lambda\right)\cdot\lambda\right]} (Eq. S1)$$

where *φ* is the photon flux (μmol m^-2^ s^-1^) measured by the sensor in the area of 400-700 nm (presented in Fig. S5), *h* is Planck’s constant (6.63·10^-34^ J s), *c* is the speed of light (3·10^8^ m s^-1^), *N_A_* is Avogadro’s constant (6.022·10^23^ mol^-1^), *λ* is wavelength (nm), *I(λ)* (W m^-2^) is light intensity at a specific wavelength and *M(λ)* (%) is the relative power of the lamp at a specific wavelength as provided by the relative spectral power distribution.

**Calculation of average irradiance spectra transmitted through the water column**

The absorbance spectra of all the different water quality solutions used *α_s_(λ)* (cm^-1^) were measured with LAMBDA 365 UV/Vis Spectrophotometer (PerkinElmer, Waltham, MA, USA) and are presented in Fig. S3. This data was used to calculate the average irradiance spectra transmitted through the water column, *I_0_(z,λ)* (W m^-2^), using the following equation (Silverman and Nelson, 2016):

$I_{0}\left( z,\lambda\right)=I_{d}(0,\lambda)\cdot\left( \frac{1-{10}^{-\alpha_{s}(\lambda)\cdot z}}{2.303\cdot\alpha_{s}(\lambda)\cdot z} \right)$ (Eq. S2)

where *I_d_(0,λ)* (W m^-2^) is the solar simulator irradiance incident spectrum on the water surface and *z* is the depth of the water column (cm). The results are presented in Fig. S4.

**Supporting figure captions**

**Fig. S1** Relative spectral power distribution of metal halide lamp used (OSRAM GmbH, Munich, Germany)

**Fig. S2** The values of photon flux measured by the sensor at different depths in the reactor filled with demineralised water (DW) or artificial flood water (FW), with *E. coli* and different concentrations of TSS. The light attenuation coefficients, *μ*, obtained from these curves are also presented

**Fig. S3** Absorbance spectra of demineralised water (DW) and artificial flood water (FW) with *E. coli* (7.8·10^6^ CFU mL^-1^) and different concentrations of TSS

**Fig. S4** Lamp irradiance spectrum and average irradiance transmitted through the water column of demineralised water (DW) and artificial flood water (FW) with *E. coli* (7.8·10^6^ CFU mL^-1^) and different concentrations of TSS

**Fig. S5** The concentration of *E. coli* in demineralised water with 200 mg TSS L^-1^, before and after filtration (11 μm) in a batch experiment in Erlenmeyer flasks

**Table S1** Comparison of TSS and *E. coli* reported concentrations in different waters with the synthetic flood water used in this research

| Water quality | TSS (mg L^-1^) | *E. coli* (CFU ml^-1^) | References |
| --- | --- | --- | --- |
| Raw municipal wastewater with minor contributions of industrial wastewater | 250 (low)  300 (medium)  600 (high) | 10^4^ (low)  5∙10^6^ (high) | Henze and Comeau, 2008 |
| Raw municipal wastewater |  | 10^6^ | Mark et al., 2015 |
| Raw influent Harnaschpolder  Artificial flood water | 29.6  0-200 | 8.5∙10^4^  10^7^ (extreme) | This research |
